# Very Low Phytoplankton Diversity in a Tropical Saline-Alkaline Lake, with Co-dominance of *Arthrospira fusiformis* (Cyanobacteria) and *Picocystis salinarum* (Chlorophyta)

**DOI:** 10.1007/s00248-019-01332-8

**Published:** 2019-02-07

**Authors:** C. Bernard, A. Escalas, N. Villeriot, H. Agogué, M. Hugoni, C. Duval, C. Carré, P. Got, G. Sarazin, D. Jézéquel, C. Leboulanger, V. Grossi, M. Ader, M. Troussellier

**Affiliations:** 1 UMR 7245 MCAM, Muséum National d’Histoire Naturelle - CNRS, 57 Rue Cuvier, CP 39, 75231 Paris Cedex 05, France; 2 UMR 9190 MARBEC, CNRS - Université de Montpellier - IRD – IFREMER, Place Eugène Bataillon, 34095 Montpellier Cedex 5, France; 3 UMR 5557 Ecologie Microbienne, Université Lyon 1 - CNRS – INRA, 69220 Villeurbanne Cedex, France; 4 UMR 7154 Institut de Physique du Globe de Paris - Sorbonne Paris Cité, Université Paris Diderot, 1 rue de Jussieu, 75005 Paris, France; 5 UMR 7266 LIENSs, Université de La Rochelle – CNRS, 2 rue Olympe de Gouges, 17000 La Rochelle, France; 6 Laboratoire de Géologie de Lyon, Université de Lyon - CNRS - UCBL - ENSL, 69220 Villeurbanne, France

**Keywords:** Phytoplankton, Diversity, Cyanobacteria, Picoeukaryote, Extreme environment, Thalassohaline lake

## Abstract

Lake Dziani Dzaha (Mayotte Island, Indian Ocean) is a tropical thalassohaline lake which geochemical and biological conditions make it a unique aquatic ecosystem considered as a modern analogue of Precambrian environments. In the present study, we focused on the diversity of phytoplanktonic communities, which produce very high and stable biomass (mean_2014–2015_ = 652 ± 179 μg chlorophyll *a* L^−1^). As predicted by classical community ecology paradigms, and as observed in similar environments, a single species is expected to dominate the phytoplanktonic communities. To test this hypothesis, we sampled water column in the deepest part of the lake (18 m) during rainy and dry seasons for two consecutive years. Phytoplanktonic communities were characterized using a combination of metagenomic, microscopy-based and flow cytometry approaches, and we used statistical modeling to identify the environmental factors determining the abundance of dominant organisms. As hypothesized, the overall diversity of the phytoplanktonic communities was very low (15 OTUs), but we observed a co-dominance of two, and not only one, OTUs, viz., *Arthrospira fusiformis* (Cyanobacteria) and *Picocystis salinarum* (Chlorophyta). We observed a decrease in the abundance of these co-dominant taxa along the depth profile and identified the adverse environmental factors driving this decline. The functional traits measured on isolated strains of these two taxa (i.e., size, pigment composition, and concentration) are then compared and discussed to explain their capacity to cope with the extreme environmental conditions encountered in the aphotic, anoxic, and sulfidic layers of the water column of Lake Dziani Dzaha.

**Electronic supplementary material:**

The online version of this article (10.1007/s00248-019-01332-8) contains supplementary material, which is available to authorized users.

## Introduction

Recently, a new saline-alkaline lake was described on Mayotte Island in the West Indian Ocean, the Lake Dziani Dzaha. The initial data obtained on this crater lake showed characteristics distinct from those reported previously for saline-alkaline lakes [1]. Located at the bottom of a volcanic crater, this small (0.2 km^2^) lake has no hydrological connection with the surrounding Indian Ocean but nonetheless its water exhibits dissolved element ratios similar to those of seawater. This lake also has some specific physico-chemical characteristics such as a completely anoxic water column under a thin euphotic surface layer (maximum depth of 1.5 m), slightly alkaline (0.23 mol L^−1^), and has high sulfide concentration (2–6 mM) below a depth of 2.2 m during the rainy season. Based on these characteristics, Lake Dziani Dzaha is considered a modern thalassohaline analogue of Precambrian environment [2]. Moreover, due to the distance of Mayotte island from the African continent (≈ 500 km), this lake can be considered as an isolated ecosystem when compared to continental saline-alkaline lakes and especially those linked by hydrological connections, such as those of the Great Rift Valley [3] or the numerous crater lakes encountered in Ethiopia [4–7], Kenya [8–12], Uganda [13–15], or South Africa [16]. Local external influences also appear limited by the characteristics of the lake’s watershed that is only constituted by the slopes of the small volcanic crater (0.9 km^2^; Mathelin [17]) without any river or permanent human settlement, but with limited food crop cultivation. Therefore, Lake Dziani Dzaha is not significantly impacted by anthropogenic activities and its hydrological characteristics are mostly dependent on the seasonal climatology, which alternates dry and rainy seasons [1].

The initial biological studies of this lake [1, 18] revealed the following: (i) the absence of macroorganisms similar to those observed in most soda-lakes (birds, fishes, invertebrates, rotifers, and cladoceres), which are known to graze phytoplanktonic communities; (ii) a large amount of phytoplanktonic biomass is produced in a stable manner; and (iii) a very low number of phytoplanktonic taxa. However, these previous characterizations of the phytoplanktonic communities were done using traditional approaches such as microscopy and strain isolation, which are now recognized to have limitations (in terms of sampling depth and culture bias) and to provide an incomplete picture of the entire community [19–21]. For instance, several recent studies based on next-generation sequencing (NGS) have shown that the phytoplanktonic communities of saline-alkaline lakes from Kenya and Ethiopia [12, 22, 23] contained a higher richness than previously estimated by microscopic methods, with a number of taxa comparable to that of other aquatic ecosystems.

In this context, we wanted to build on the results from the above-mentioned study of Lake Dziani Dzaha and further characterize the phytoplanktonic community of this lake using a combination of NGS and more classical tools such as microscopy and flow cytometry (FCM).

Doing so, we want to answer the following questions: (i) What is the richness and the composition of the phytoplanktonic community in the Lake Dziani Dzaha? More specifically, we want to determine whether rare taxa exist, as reported in most, if not all, saline alkaline ecosystems, or if they are excluded due to the isolation of the lake and limited environmentally suitable niche for photosynthetic organisms. (ii) Do the drastic abiotic characteristics (high salinity, high pH, limited euphotic depth, anoxia, and sulfidic layer) coupled with high nutrient concentrations and the absence of large grazers lead to the dominance of only one phytoplanktonic species as would be expected according to ecological theories such as the “Killing the winner” hypothesis [24] or the “competitive exclusion” principle [25]?

## Material and Methods

### Study Site, Sampling, and Environmental Parameters

**Fig. 1:**
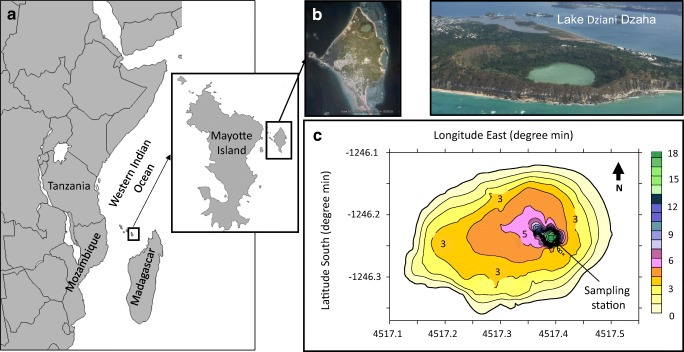
Lake Dziani Dzaha (Mayotte Island) is an isolated, round crater lake with an area of approximately 0.25 km^2^ (**a**, **b**). The bathymetric map showed a mean depth of approximately 4 m and an 18-m deep pit, located in the eastern part of the lake (**c**). **b**, right picture: ©M. Troussellier, CNRS

Lake Dziani Dzaha is a volcano crater lake located in Mayotte Island of the Comoros Archipelago, West Indian Ocean (12° 46′ 15.6″ S; 45° 17′ 19.2″ E). The lake surface area is approximately 0.25 km^2^ with a mean depth of approximately 4 m and a narrow 18-m-deep pit, located in the eastern part of the lake (Fig. 1). This tropical crater lake is characterized by high salinity (up to 70 psu) and alkalinity (0.23 mol L^−1^), conductivity between 77.1 and 79.7 mS and pH between 9 and 9.5 [1]. The lake is very turbid (Secchi transparency < 0.15 m), and the concentration of chlorophyll *a* (Chl*a*) is very high (up to 875 μg L^−1^ in 2010–2011). The lake is also characterized by the absence of zooplankton, fishes, and lesser flamingos [1]. Actively growing stromatolites have been observed in the shallow waters of the lake shores [26].

Four sampling campaigns were conducted in April and October 2014 and in April and November 2015. The precipitation data for the corresponding sampling campaigns were obtained from the Meteo-France Meteorological Observation Station located near Lake Dziani Dzaha (http://www.infoclimat.fr). Discrete water sampling was carried out at the deepest point of the lake (18 m) (Fig. 1) using a horizontal 1.2-L Niskin bottle. Water samples were collected at 16 depths (0, 0.25, 0.5, 0.75, 1, 1.5, 2.5, 3.5, 5, 7, 9, 11, 13, 15, 17, 18 m) and the samples for biological and chemical analyses were processed in the field laboratory within 2 h. The vertical profile of salinity/conductivity, temperature, pH, dissolved O_2_, and redox potential were performed using YSI 6600 (CTD-O_2_-pH-redox) (YSI, Yellow Springs, OH, USA) and/or SDOT nke (O_2_ optode). The attenuation coefficient *K*_*d*_ was determined from discrete PAR measurement profiles using a SPAR nke (PAR). The total alkalinity was determined by Gran titration [27]. The content of soluble sulfide (∑S(-II); H_2_S), ammonium and ammonia (∑N(-III); NH_4_), and soluble-reactive phosphorus (SRP; PO_4_^3−^) was determined colorimetrically in the field lab using the Aqualytic SpectroDirect spectrophotometer and Merck reagent kits. The concentration of Chl*a* was analyzed after extraction using 96% ethanol by ultra-sonication in an ice bath for 30″, and further extraction was allowed overnight at 4 °C in dark. The extract was filtered and the filtrate was analyzed spectrophotometrically at 400–750 nm. The concentration of Chl*a* was calculated according to Ritchie [28].

### Cell Abundance

#### Picophytoplankton

Cell abundances were estimated for all the sampling depths, except for the 18-m depth, which contained a lot of sediment particles that disturb the flow cytometric analysis. Sub-samples (1.6 mL) of the lake water samples from each depth were preserved using 0.2-μm filtered formaldehyde solution (2% final concentration) and stored in liquid nitrogen (− 196 °C). They were further stored at − 80 °C before analysis using a FACSAria Flow cytometer (Becton Dickinson, San Jose, CA, USA) equipped with the HeNe air-cooled laser (633 nm, 20 mW). The cells excited at 633 nm were detected and enumerated according to their forward-angle light scatter (FALS) and right angle light scatter (RALS) properties, and red fluorescence from phycocyanin (660/20 nm) and chlorophyll (675/20 nm) pigments. Fluorescent beads (1–2 μm) were systematically added to each sample. True count beads (Becton Dickinson, San Jose, CA, USA) were added to determine the volume analyzed. List-mode files were analyzed using the BD FACSDiva software. This method discriminates autotrophic picoeukaryote by using their chlorophyll pigments [29]. Pure culture of *Picocystis salinarum* ALCP 144.1 (Algothèque Laboratoire Cryptogamie Paris, Museum National of Natural History) was used to confirm the flow cytometric signature of this autotrophic group.

#### Cyanobacteria

Cell abundance was estimated for the same sampling depths than for picophytoplankton. Sub-samples (9 mL) of the lake water samples were collected at each sampling depth for microscopic examination, identification, and measurement of cyanobacterial cells. The samples were fixed with 5% formaldehyde and the taxa were identified as described by Cellamare et al. [18]. The cyanobacterial count data were obtained by the Utermöhl method using an Eclipse TS100 inverted microscope at ×600 magnification (Nikon Instruments Inc., Melville, NY, USA) as described in Catherine et al. [30].

### Functional Traits of Isolated *Arthrospira fusiformis* and *Picocystis salinarum* Strains

#### Culture Conditions and Cells Counting

Six clonal non-axenic strains of *A. fusiformis* PMC 851.14, 894.15 and 917.15 (Paris Museum Collection) and *P. salinarum* ALCP 144.1, 145.1 and 146.1, isolated from Lake Dziani Dzaha were used. Each strain was grown as batch cultures in 250-mL Erlenmeyer flasks containing 200 mL of Z8-salt medium [31], at 25 °C under 30 μmol photons m^−2^ s^−1^ provided by daylight fluorescent tubes (OSRAM Lumilux®, München, Germany), with a 16:8-h light/dark photoperiod. Sampling was performed at the end of exponential growth phase. The cell abundance was estimated by Malassez-based method for each culture as described by Catherine et al. [32]. Three replicates were counted for each culture.

#### Cell Size Estimation

Length of *Arthrospira* filaments were measured and counted by the Utermöhl method using an inverted microscope at ×600 magnification. Cell number per filament was then counted using an upright microscope (on at least 50 filaments), at ×1000 magnification. We applied the mean of cell number per filament length to each sample and *Arthrospira* filaments were then converted to cell abundance (mL^−1^). This method is described in the SOP Cyanobacterial samples: preservation, enumeration, and biovolume measurements [32].

#### Cell Pigment Concentrations

Four milliliters of each culture replicate were used for phycocyanin analysis as described by Yéprémian et al. [33]. For lipophilic pigments, 18 mL of each culture was filtered using GF/F Whatman filters, extracted with methanol, and analyzed according to Ras et al. [34]. The results are expressed as the amount of each pigment by cell volume (fg μm^−3^). The cell volume of the two species was computed from their respective sizes [18] using models for the specific cellular shape of each species [35].

### DNA Extraction and Illumina Sequencing

NGS analysis was performed on samples collected at 0.5, 1, 2.5, 5, 11, 15, and 17 m depths. Water subsamples (20 mL) were filtered through 3- and 0.2-μm pore-size polycarbonate filters (Millipore, pressure < 10 kPa), and then stored at − 20 °C until nucleic acid extraction. The DNA was extracted separately from both the filters, using the Power Water DNA isolation kit (MoBio Laboratories) as described by the manufacturer for maximum yield. The DNA quality was analyzed by 1% (*w*/*v*) agarose gel electrophoresis and quantified using NanoDrop. The amplification of bacterial V3–V5 region of the 16S rRNA genes was performed in triplicate, using the universal primers 357F [36] and 926R [37] allowing the amplification of bacteria and eukaryotic chloroplast sequences, respectively. Additionally, the DNA was extracted without any biological matrix, and it was considered as a negative control to evaluate ambient contamination. High-throughput sequencing was achieved after pooling the PCR-triplicates and a multiplexing step, by HiSeq Rapid Run 300 bp PE technology using the Illumina HiSeq 2500 system (GATC Biotech, Konstanz, Germany).

### Sequence Processing

The 16S rRNA paired-end reads obtained from both the filters were merged with a maximum of 10% mismatches in the overlap region using FLASh [38]. The denoising procedure was carried out by discarding reads containing ambiguous bases (N) or reads outside the range of expected length (e.g., 450–580 bp). After dereplication, the sequences were clustered using SWARM [39] that uses a local clustering threshold. Chimeric sequences were removed using VSEARCH [40]. Furthermore, the sequences representing < 0.005% of the total number of sequences [41] along with singletons were also removed. A preliminary taxonomic affiliation was performed with both RDP Classifier [42] and BLASTN+ [43] against the 128 SILVA database [44]. This procedure was automated in the FROGS pipeline [45]. The contaminant operational taxonomic units (OTUs) identified from the control samples were removed.

### Taxonomic and Phylogenetic Affiliation

The affiliation of the phytoplanktonic OTUs was carried out using the National Center for Biotechnology Information Basic Local Alignment Search Tool (NCBI BLAST) tool on three databases: (i) GenBank sequence database (NCBI 1998), (ii) sequences of cyanobacterial PMC strains isolated from Lake Dziani Dzaha [18], and (iii) sequences in the PhytoRef database [46]. A phylogenetic approach was then used to create a 16S rRNA gene phylogeny with selected sequences based on the first affiliation obtained by similarity analysis (NCBI BLAST) and the reference databases (Genbank, PMC strains, and PhytoRef). Phylogenetic reconstruction was performed using the MEGA6 software with 1000 iterations, and according to three methods: neighbor joining (NJ), maximum likelihood (ML), and maximum parsimony (MP). The outgroups were selected based on the results of similar studies: (i) *Rhodopseudomonas palustris* for cyanobacteria and (ii) *Aulacoseira ambigua* (Ochrophyta), (iii) *Vitrella brassicaformis* (Myzozoa), and (iv) *Euglena gracilis* (Chlorophyta) for photosynthetic eukaryotes [47].

### Data Analyses

Partial least square (PLS) regression was used to identify the environmental factors (Xi) affecting the abundance of *P. salinarum* and *A. fusiformis* (Yi), This method is particularly suited for analysis of data with several explanatory variables (Xi) and a limited number of observations (Yi), and in cases where there is multicollinearity among analyzed variables. In our case, we applied PLS as the data points were collected along a gradient and constituted non-statistically independent observations which resulted in significant correlations across the potential explanatory variables (such as temperature, salinity, H_2_S, and NH_4_^+^) [48, 49]. All the analyses were performed using the XLSTAT-Ecology software (Addinsoft, Paris, France).

## Results

### Environmental Characteristics of the Water Column

**Fig. 2:**
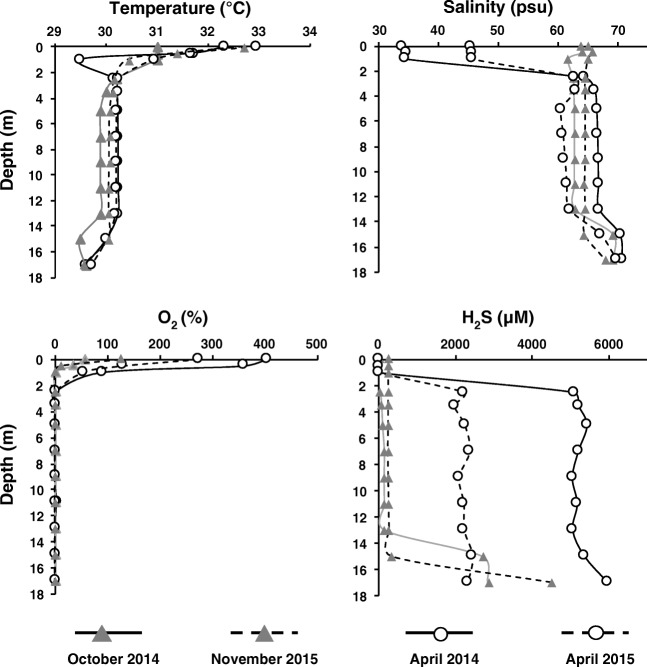
Changes in the main physico-chemical characteristics (temperature, salinity, soluble sulfide (H_2_S), and oxygen saturation (%O_2_) as a function of depth and sampling campaign. April 2014 and 2015: stratified water column, end of the rainy season. October 2014 and November 2015: non-stratified period, end of the dry season

All four campaigns were characterized by high PAR values (2539–2787 μM m^−2^ s^−1^) at the lake surface. There were significant differences in rainfall between seasons: 1138–1056 mm during the rainy season (2014–2015, from November to March) and 53–154 mm during the dry season (2014–2015, from April to October). Several physico-chemical characteristics were clearly identified depending on the season and depth, along the water column (Fig. 2). The water column exhibited two distinct physico-chemical profiles depending on the sampling period:

- At the end of the rainy season (April 2014 and 2015), we observed a stratified water column with two distinct layers well separated by physical and chemical clines. The surface layer (≤ 2.2 m) was characterized by weak light penetration (*K*_*d*_ = 11.6–12.7 m^−1^), temperatures between 30 °C and 33 °C, high level of oxygen saturation (15%–400%), low level of H_2_S (≤ 100 μM), high pH values (> 9.5 in 2014 and > 9.2 in 2015), and moderate salinity (35–45 psu). The intermediate layer (2.2–16 m) was characterized by the absence of light penetration, no oxygen, lower pH (9.5–8.9 in 2014 and 9.0–8.7 in 2015), and constant salinity (≈ 65 psu). Low NH_4_^+^ and PO_4_^3−^ concentrations (15 ± 14 and 23 ± 38 μM, respectively) were observed in the surface and intermediate layers during the stratified season.
- At the end of the dry season (October 2014 and November 2015), light penetration was weak (*K*_*d*_ = 18.4 m^−1^), temperature was high, and oxygen saturation was observed at the surface of the lake (≤ 2.2 m). Furthermore, the salinity, pH, and H_2_S clines disappeared leading to a non-stratified water column (salinity ≈ 65 psu, pH ≈ 9.0, H_2_S ≈ 200 μM), from the surface to the deep permanent layers. Strong gradients of inorganic nutrients were also observed in the lake column, with low NH_4_^+^ and PO_4_^3−^ concentrations (15 ± 14 and 23 ± 38 μM, respectively) in the intermediate layer and very high and variable NH_4_^+^ and PO_4_^3−^concentrations (2653 ± 2133 and 172 ± 114 μM, respectively) in the intermediate and bottom layers.

During both seasons and all the sampling campaigns, a deep layer characterized by no light penetration, no oxygen, high salinity (≈ 70 psu), low pH values (< 9.0), and very high H_2_S concentration (> 2000 μM) was observed at the bottom of the water column (14–17 m).

Phytoplankton biomass, estimated by Chl*a* concentration, also exhibited vertical gradients, with higher and less variable values observed in the upper layers (mean_2014–2015_ = 652 ± 179 μg L^−1^) than those in the bottom layers (mean_2014–2015_ = 171 ± 201 μg L^−1^).

### Composition and Structure of Phytoplanktonic Communities

#### Taxonomic and Phylogenetic Affiliation of Phytoplanktonic OTUs

Using 16S rRNA genes amplicon, we obtained 455 16S rRNA-OTUs including bacteria, cyanobacteria, and eukaryotic plastids. The phytoplanktonic community was composed of 15 OTUs, with eight OTUs being affiliated to cyanobacteria and seven OTUs to eukaryotic plastids (Tables 1, S1, and S2).

**Table 1:** Affiliation of cyanobacterial taxa and photosynthetic eukaryotes OTUs ranked in decreasing order sequence numbers. Their relative abundance (%) in the total phytoplanktonic community, either cyanobacteria or photosynthetic eukaryotes communities, were also reported

		Proportion that form the sequences of each phytoplanktonic taxa in different communities		
Taxa	Number of sequences	Phytoplankton (%)	Cyanobacteria (%)	Eukaryotic phytoplankton (%)
*Arthrospira fusiformis*	8,249,182	84.78	99.99	–
*Sodalinema komarekii*	99	1.02 10^−3^	1.20 10^−3^	–
*Leptolyngbya* sp. 1	66	6.78 10^−4^	8.00 10^−4^	–
*Synechococcus* sp.	50	5.14 10^−4^	6.06 10^−4^	–
*Leptolyngbya* sp. 2	31	3.19 10^−4^	3.76 10^−4^	–
*Xenococcus* sp.	9	9.25 10^−5^	1.09 10^−4^	–
*Leptolyngbya* sp. 3	2	2.06 10^−5^	2.42 10^−5^	–
*Leptolyngbya* sp. 4	1	1.03E-05	1.21 10^−5^	–
∑ cyanobacteria	8,249,440	84.78	**–**	**–**
*Picocystis salinarum*	1,480,251	15.21	–	99.99
*Kryptoperidinium* sp.	100	1.03 10^−3^	–	6.76 10^−3^
*Cymbellales* sp. 1	9	9.25 10^−5^	–	6.08 10^−4^
*Cymbellales* sp. 2	7	7.19 10^−5^		4.73 10^−4^
*Cymbellales* sp. 3	3	3.08 10^−5^	–	2.03 10^−4^
*Naviculaceae* sp.	2	2.06 10^−5^	–	1.35 10^−4^
*Gyrosigma* sp.	1	1.03 10^−5^	–	6.76 10^−5^
∑ eukaryotic phytoplankton	1,480,373	15.21	**–**	**–**
Total	9,729,813			

Each cyanobacterial OTU was affiliated either to the genus or species level (Tables 1 and S1). The phylogeny of cyanobacteria (Fig. S1) showed that the eight observed OTUs belonged to the orders Oscillatoriales (*A. fusiformis*), Synechococcales (Family Synechococcaceae: *Synechococcus*, Family Leptolyngbyaceae: *Sodalinema komarekii* and *Leptolyngbya* spp. 1 to 4), and Pleurocapsales (*Xenococcus* sp.). Among these eight OTUs, two species (*A. fusiformis* and *S. komarekii*) have been already described in the isolated strains from Lake Dziani Dzaha by a polyphasic approach [18].

The seven eukaryotic plastid-related OTUs (Table S2) were distributed to three phyla (Fig. S2): (i) Ochrophyta, including three OTUs affiliated to the Cymbellaceae and Cymbellales (sp. 1 to 3), but the bootstrap scores did not support the inclusion of these OTUs to any genus or species and two OTUs to the Naviculaceae (sp.) and Naviculales (*Gyrosigma* sp.), (ii) Myzozoa, with one OTU closely related to *Kryptoperidinium foliaceum* within the order Peridiniales, and (iii) Chlorophyta, with one OTU affiliated to *P. salinarum* that forms a separate phylogenetic lineage within Prasinophyceae [14, 50].

### Contribution of Cyanobacteria and Photosynthetic Eukaryotes to the Diversity of 16S rRNA Genes

Although the phytoplanktonic community represented only 3.3% of the total observed richness (15 OTUs among the 455 OTUs in the total dataset, data not shown), it nonetheless represented 52% of the total number of sequences (18,747,809 sequences). *A. fusiformis* (8,249,182 sequences) and *P. salinarum* (1,480,251 sequences) were the two most abundant taxa of the planktonic community in Lake Dziani Dzaha. Among the photosynthetic microorganisms, *A. fusiformis* and *P. salinarum* were, by far, the only dominant microorganisms, representing 99.99% of the total number of cyanobacterial and photosynthetic eukaryote sequences, respectively (Table 1). Photographs of these two dominant OTU representatives are presented in Fig. S3.

### Changes in the Abundance of the Dominant Phytoplanktonic Taxa along the Water Column

The over-dominance of only two phytoplanktonic, *P. salinarum* and *A. fusiformis*, provided an opportunity to easily and accurately estimate their abundance by microscopy and flow cytometry, respectively. The observed mean abundances were high, with 1.44 × 10^6^ and 7.78 × 10^5^ cells mL^−1^ for *A. fusiformis* and *P. salinarum*, respectively (Table 2). Taking into account all the samples, the standardized number of sequences of these two taxa were significantly related to their abundance values estimated using FCM (Fig. S4). The relationship was linear for *Picocystis* but asymptotic for *Arthrospira*, probably as a consequence of the finite standardized number of analyzed sequences versus theoretically limitless number of cells counted using FCM.

**Table 2:** Mean and dispersion statistics of the abundances (cells mL^−1^) estimated using flow cytometry for *P. salinarum* and microscopy for *A. fusiformis*

Taxa	Number	Mean	Min	Max	SD	CV (%)
*P. salinarum*	52	7.78 10^5^	3.15 10^4^	2.67 10^6^	6.67 10^5^	85.7
*A. fusiformis*	42	1.44 10^6^	6.48 10^4^	4.66 10^6^	1.05 10^6^	73.1

**Fig. 3:**
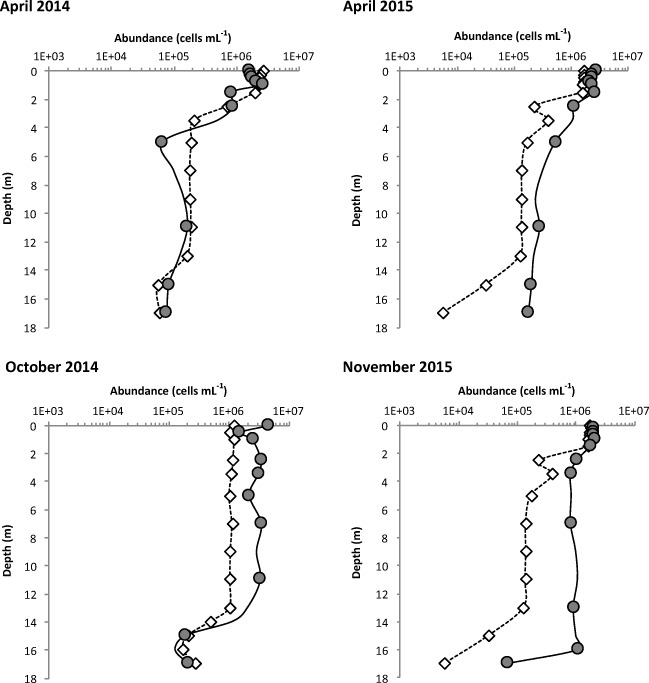
Changes in the abundance of *Picocystis salinarum* (open squares) and *Arthrospira fusiformis* (gray circles) as a function of depth during the four sampling campaigns. The abundance of *P. salinarum* and *A. fusiformis* was estimated by flow cytometry and microscopy, respectively

The maximum values were recorded in the upper euphotic layer (Fig. 3; *P. salinarum*: 2.67 × 10^6^ cells mL^−1^, April 2014; *A. fusiformis*: 4.66 × 10^6^ cells mL^−1^, October 2014).

Large variations in the abundance of species were observed with depth (Fig. 3), with both the taxa exhibiting a decreasing trend with depth (Spearman rho = − 0.554, *P* < 10^−3^ for *A. fusiformis* and − 0.665, *P* < 10^−4^ for *P. salinarum*). However, we observed differences in these trends according to the stratification state of the water column (Fig. 3). During the stratified period (April 2014 and 2015), the abundance of both taxa showed a quasi-exponential decrease below the thin euphotic layer. During the non-stratified period (October 2014 and November 2015), small variations in the abundance of the two taxa were observed, notably between the surface and 14–16 m bottom layers, where the physico-chemical characteristics of the water column were homogeneous. Below this limit, the abundance of both the species exhibited a significant decrease. The relative decrease in taxa abundance between the top and bottom layers of the water column was very high and constant for *A. fusiformis* (93%–97%) and highly variable for *P. salinarum* (73%–99%). The lowest decrease in the abundance of *P. salinarum* was observed during the non-stratified period (78% in October 2014 and 73% in November 2015).

### Relationships between the Abundance of the Dominant Photosynthetic Taxa and Physico-Chemical Characteristics of the Water Column

The results of the PLS regression models are provided in Table 3. The *R*^2^ associated with different models were very high, with higher values for *P. salinarum* than for *A. fusiformis* model. Among the four tested explanatory variables, temperature was the only variable that positively affected the abundance of *P. salinarum* and *A. fusiformis*, and showed relatively low standardized regression coefficient. The three other variables (salinity, log H_2_S, and log NH_4_^+^) negatively correlated with phytoplankton abundance. During the stratified and the non-stratified seasons, the explanatory variable with the highest coefficient value was salinity for *P. salinarum*, whereas, it was log H_2_S for *A. fusiformis*.

**Table 3:** Results from partial least squares (PLS) regression performed between the abundances of *P. salinarum* and *A. fusiformis* and the tested environmental variables (T = temperature, S = salinity, H_2_S = H_2_S concentration, NH_4_^+^ = NH_4_^+^ concentration). n = number of data. *R*^2^ = determination coefficient. Stratified period: April 2014 and 2015. Non-stratified period: October 2014 and November 2015

	Stratified period		Non-stratified period	
Taxa	*P. salinarum*	*A. fusiformis*	*P. salinarum*	*A. fusiformis*
*n*	30	30	29	29
*R* ^2^	0.885	0.781	0.866	0.580
*T*	+ 0.213	+ 0.225	+ 0.138	+ 0.134
*S*	− 0.336	− 0.275	− 0.324	− 0.250
Log H_2_S	− 0.314	− 0.333	− 0.317	− 0.278
Log NH_4_^+^	− 0.264	− 0.245	− 0.295	− 0.227

### Pigment Composition of *P. salinarum* and *A. fusiformis* Isolated Strains

The results of the analysis of different pigment classes of the strains *P. salinarum* and *A. fusiformis* are presented in Table 4. *Picocystis salinarum* cells exhibited a higher diversity in their pigment composition than *A. fusiformis* (nine and five, respectively). Among the accessory pigments, specific carotenoids were exclusively detected in *P. salinarum* (neoxanthin, violaxanthin, antheraxanthin, and two unknown lipophilic pigments). Specific pigments also existed in *A. fusiformis* cells, including phycocyanin and two lipophilic pigments (zeaxanthin and an unknown pigment). Expressed as the amount of pigment by cellular volume unit, *P. salinarum* cells presented higher concentrations of both Chl*a* and *b* (51.9 ± 12.8 and 21.1 ± 5.8 fg μm^−3^, respectively) than those of *A. fusiformis* (6.0 ± 4.9 and 0 fg μm^−3^, respectively). On the contrary and as expected, *A. fusiformis* had a high content of phycocyanin (51.1 ± 51.8 fg μm^−3^), which *P. salinarum* does not have.

**Table 4:** Pigments composition and concentration (fg μm^−3^) of *Arthrospira fusiformis* (Cyanobacteria) and *Picocystis salinarum* (Chlorophyta) strains isolated from Lake Dziani Dzaha. The specific absorption wavelengths (nm) was indicated for each pigment. ALCP = Algothèque Laboratoire Cryptogamie Paris. PMC = Paris Museum Collection, Museum National of Natural History

Pigments (fg μm^−3^)		Absorption	*P. salinarum*	*P. salinarum*	*P. salinarum*	*A. fusiformis*	*A. fusiformis*	*A. fusiformis*
		(nm)	ALCP 144.1	ALCP 145.1	ALCP 146.1	PMC 851.14	PMC 894.15	PMC 917.15
Carotenoids	Neoxanthin	438–466	3.0	2.1	1.7	0	0	0
	Violaxanthin	443	0.4	0.4	0	0	0	0
	Antheraxanthin	449–452	10.2	7.3	5.0	0	0	0
	Zeaxanthin	400–500	0	0	0	0.2	0.6	0.2
	Lutein	445–473	4.2	3.0	5.2	0	0	0
	β-Carotene	452	2.7	1.8	1.9	0.3	1.1	0.5
	Chlorophyll b	445, 645	27.5	19.5	16.2	0	0	0
Chlorophylls	Chlorophyll a	430, 660	66.5	46.0	43.1	2.7	11.6	3.7
	Phaeophytin a	666	0.3	0.2	0	0	0	0
Phycobiliprotein	Phycocyanin	500–650	0,0	0.0	0.0	24.4	110.8	18.1
Unknown	Unknown_x1		0.7	0.6	0.9	0	0	0
	Unknown-x2		1.6	1.2	3.8	0	0	0
	Unknown_x3		0	0	0	0.1	0.4	0.2

## Discussion

### Drastic Environmental Changes in the Lake Dziani Dzaha Pelagic Ecosystem

Lake Dziani Dzaha is a tropical thalassohaline crater lake, with unique specific physico-chemical characteristics. The water of the Dziani Dzaha Lake probably originates from the nearby Indian Ocean with further modifications of its physico-chemical characteristics by precipitations, hydrothermal activity, precipitation of dissolved elements, and biogeochemical activity. This marine origin is original compared with that of most known other alkaline and saline lakes that are, for the great majority, inland water bodies whose salinity is due to dissolved continental minerals [1].

The hydrological characteristics of Lake Dziani Dzaha are mostly dependent on the seasonal climatology, which is based on alternating dry and rainy seasons. Low surface salinity and a halocline at a depth of 2.2 m can be attributed to the heavy rainfalls occurring during the rainy season in Mayotte Islands. During dry season, evaporation progressively increased the salinity of the surface layer, easing the mixing of water column and disappearance of the halocline. Therefore, the lake can be considered a monomictic lake (mixing once a year during the southern hemisphere winter) with special features in terms of oxygen content, i.e., permanent anoxia at a depth of approximately 2 m even in the absence of halocline. In summary, the water column in the deepest part of the lake undergoes important changes in its physicochemical characteristics, thus, defining several layers along the water column during both the seasons. These contrast layers, based on the physico-chemical characteristics, were observed between seasons from 2010 to 2015 [1] and this study. They define several environmental niches, which might have strong effects on phytoplanktonic diversity and distribution, along the water column.

The estimated phytoplanktonic biomass, expressed as mean chlorophyll *a* concentrations, was very high in 2014 (566 μg Chl*a* L^−1^) and 2015 (692 μg Chl*a* L^−1^), and in the same range than the ones already observed in 2010 (685 μg Chl*a* L^−1^) and 2011 (702 μg Chl*a* L^−1^) [1].

### Diversity and Composition of Phytoplanktonic Community

In the present study, the richness of phytoplanktonic community in the lake was low, with only 15 taxa being detected, eight of them belonging to cyanobacteria and seven to photosynthetic eukaryotes. To the best of our best knowledge, no study on the phytoplanktonic diversity of thalassohaline lake have been carried out with metabarcoding and metagenomic approaches. The richness of phytoplanktonic communities of soda lakes located in the Kenyan and Ethiopian Rift Valleys, which are the most studied aquatic continental ecosystems close to Lake Dziani Dzaha, have been mainly evaluated through microscopic identification [5, 51–53]. The few studies based on next-generation sequencing (NGS) on the microbial communities of these lakes have focused on prokaryotic communities [12, 54]. The only NGS-based study of both prokaryotic and eukaryotic organisms in several Ethiopian Lakes was by Lanzén et al. [22]. However, the number of phytoplanktonic taxa that can be retrieved from their database was considerably lower than that of other studies on the same lakes but based on microscopy [5, 55].

Considering the microscopy-based richness estimation of phytoplanktonic communities of Kenyan and Ethiopian lakes, which range between 72 (Koka Lake; [5]) and seven (Abijata Lake; [5]), the richness of phytoplanktonic community in Lake Dziani Dzaha was in the lower range and it is among the lowest recorded in the saline-alkaline lakes in this part of the world. Among the crater lakes located in the same geographical and climatic area, a single study in Lake Oloidien (Kenya) reported the characterization of phytoplanktonic communities using NGS [12]. These authors observed up to 30 cyanobacteria and more than 15 eukaryotic taxa. However, Lake Oloidien differs greatly from Lake Dziani Dzaha in terms of salinity, which remains quite lower and more variable (< 4 ppt), the presence of zooplankton and the non-permanent flamingo populations, along with permanent human populations that rely on the lake water for different uses.

Among the 15 phytoplanktonic OTUs, the two significantly dominant ones were *A. fusiformis* and *P. salinarum*; both the species being commonly described as dominant phytoplanktonic taxa in equatorial soda lakes [54, 56–58]. Among the rare cyanobacteria OTUs, one corresponds to a new species (*S. komarekii*), which has been recently described in strains isolated from stromatolites, and it might be considered endemic to Lake Dziani Dzaha [18]. The other rare cyanobacterial OTUs (*Leptolyngbya* spp. 1–4, *Synechococcus* sp., and *Xenococcus* sp.) have also been reported with relatively high frequency in the stromatolites of the lake [26]. The fact that all these cyanobacterial species were detected with a very low frequency in the water column and a high frequency in stromatolites, implies that the preferential habitat of these species is probably the stromatolites, where Cyanobacteria have been shown to influence their shape and mineralogy [26].

With respect to the rare eukaryotic plastid-related OTUs, only *Kryptoperidinium* sp. was detected in stromatolites with a higher frequency than that in the water column [59]. Regarding Bacillariophyta, the assignation level of Cymbellaceae spp. clusters (Family) does not allow comparison with the composition of diatoms from other aquatic ecosystems. However, both Cymbellaceae species and *Gyrosigma* sp., affiliated with *Gyrosigma fasciola*, have already been observed in the Mozambique Channel [60]. *Gyrosigma* sp. has never been reported as a typical species of soda or thalassohaline lakes. Thus, it can be hypothesized that most of the rare eukaryotic species detected in Lake Dziani Dzaha are probably of marine origin. Finally, some common thalassohaline or soda lakes taxa (e.g., Chlorophyta such as *Kirchneriella*, *Monoraphidium*, *Raphidocelis*, *Selenastrum*, and *Tetranephris* [23] were not detected in Lake Dziani Dzaha.

Overall, these results support our initial hypothesis of very low taxonomic richness of phytoplanktonic community in the Lake Dziani Dzaha pelagic ecosystem. However, the two-dominant species (*A. fusiformis* and *P. salinarum*) were the same as those reported in most saline-alkaline lakes, whereas, the very rare OTUs observed in the water column appeared to originate either from stromatolites or surrounding marine environment. Different and complementary processes can explain the low richness. First, the geographical isolation of this insular crater lake from other alkaline-saline lakes avoids or significantly limits the movement of phytoplanktonic species, which might occur in continental lakes through dispersal agents (such as water, air, animal, and human) [61]. Second, the small size of the lake coupled with drastic environmental conditions in the water column limits the suitable niches for phytoplankton to the thin upper layer of the water column. Third, a main difference from other alkaline or thalassohaline lakes is the absence of grazers (zooplankton, fishes, and birds such as flamingos [62]). The absence of top-down control of phytoplanktonic populations might allow dominant species to establish and persist [63, 64]. Fourth, the large amount of phytoplanktonic biomass suggests that there is no nutrient limitation and implies a strong light attenuation, resulting in a competition for light among phytoplanktonic species leading to the dominance of the best competitors [65].

### Only One Prokaryotic and One Eukaryotic Taxa Dominated the Phytoplanktonic Community from the Surface to Bottom Layers of the Water Column

Considering that the above-mentioned processes can explain the observed very low diversity, both competitive exclusion [66] and killing the winner theories [24, 67] predict that only one species can dominate and exclude the others. However, in Lake Dziani Dzaha, we observed the co-dominance of two distinct species in the same environmental niche, which, to the best of our knowledge, has not been reported in saline-alkaline lakes.

A first explanation of this unexpected result might be that co-occurrence is possible because of the distinct capacities of the two taxa to cope with light-limitation (Tables 4 and S3). Light limitation mostly refers to the quantitative aspect, but light is not a single resource, such as nutrients (e.g., NH_4_^+^ and PO_4_^3−^). Light is composed of different wavelengths, and photosynthetic organisms have developed adaptations to use different regions of the photosynthetically active radiation (PAR) through a diverse range of pigments. Recently, Burson et al. [68] proposed an explanation for the unexpected co-occurrence of different phytoplanktonic species, where the best competitor for light is expected to dominate the phytoplanktonic community. In the two dominant taxa of Lake Dziani Dzaha, differences in their pigment composition were obvious (Table 4). For instance, if they share the ubiquitous Chl*a* pigment with absorption peaks at 440 and 680 nm (blue and red regions of the spectrum), *P. salinarum* cells can exhibit higher concentration of Chl*a* than *A. fusiformis*, given the potential better efficiency of *P. salinarum* to capture energy associated with Chl*a* absorption wavelengths. The cyanobacteria *A. fusiformis* contains the accessory pigment phycocyanin, which has an absorption peak at 630 nm (orange region), whereas, *P. salinarum* contains Chl*b* with an absorption peak at 475 (blue-green region) and 650 nm (red region). Furthermore, *P. salinarum* also contains several other pigments, including diatoxanthin (451 and 479 nm) and monadoxanthin (448 and 475 nm), which enables them to efficiently use the blue-green part of the light spectrum.

A second explanation of the non-competitive access to light resources by these two taxa in the thin surface layer of the water column might be the significant difference in their size. To prevent sinking, the large filaments of *A. fusiformis* benefit from intracellular aerotopes, which regulate their buoyancy [69]. Due to their very small size, *P. salinarum* cells have a very low intrinsic sinking rate [70] that can be lowered by the viscosity of lake water where salinity is higher than that of seawater. Thus, these two taxa exhibited distinct but non-competitive approach to remain in the upper part of the euphotic layer, where they attained the maximum abundance.

Coupled with the low sinking property they confer to the phytoplanktonic cells [71], high surface/volume ratio (Table S3 and Fig. S3), as in picophytoplanktonic cells, compared with that of nano- or microphytoplanktonic cells, has also been proposed as a way to cope with light limitation [72]. In the present study, these two co-dominant taxa were also observed and enumerated in all the samples by both microscopy and flow cytometry. The abundance of these two-species showed that they mostly co-occur and exhibit a significant decrease with depth, especially when the water column is stratified. However, if we hypothesize that most, if not all, the cells of both taxa were produced in the top layer of the lake and then sank, it is remarkable that during non-stratified periods, approximately 25% of *Picocystis* cells were able to survive in the extreme hostile physico-chemical environments that prevail in the bottom layers of the water column. Contrarily, and irrespective of the water column structure, only a small percent of abundance of *Arthrospira* cells in the top layer was observed in these bottom layers. To the best of our knowledge, such differential behavior has not been reported by the studies on soda lakes (Table S3).

### Factors Regulating the Dynamics of the Two Dominant Phytoplanktonic Taxa

As mentioned previously, it is probable that high PAR in the upper layer allowed active growth of the two photosynthetic taxa, but they also seem to maintain minimum growth when the PAR decreased. The ability to grow in low-light environments has been demonstrated for *P. salinarum*, with a positive growth at very low irradiance (0.6 μM photons m^−2^ s^−1^), showing its high potential for photoacclimatation and shade-adapted photosynthesis [73]. The high Chl*a* pigment concentration, as observed in the isolated strains of the present study, might also help this species to optimize its ability to cope with very low light intensity [74]. Similarly, specific adaptive mechanisms to very low irradiance have been developed by *Arthrospira*, which can increase the cellular pigment concentrations [75].

From the upper layer to the bottom of the water column, the results of statistical modeling showed that the abundance of the two over-dominant taxa were significantly correlated with all the tested environmental factors. The abundance of *P. salinarum* and *A. fusiformis* positively correlated with the temperature. Temperatures of near or over 30 °C has been shown to potentially favor the growth of the two taxa [73–76]. The negative relationships between the abundance of the two taxa versus salinity, H_2_S, and NH_4_^+^ indicated that below the upper layer, they were both subjected to adverse conditions.

The negative relationship with H_2_S can result from the effect of anoxic conditions and/or the direct toxic effect of H_2_S. These two taxa might have different ways to cope with high levels of H_2_S, but the underlying mechanisms remain to be elucidated.

Growth and photosynthetic activity of *P. salinarum* strains were observed under anoxic conditions and after treatment with 100 μM Na_2_S with comparable rates than those measured under oxic conditions [73]. The results suggested that the ability to maintain oxygenic photosynthesis even under low light condition might allow the proximal environment of *P. salinarum* cells to be suboxic, thus, reducing the redox stress. However, below the surface layer of the lake, no light was available for photosynthesis and during the stratified season the H_2_S concentration was significantly above 1000 μM. The significant abundance of *P. salinarum* in the bottom layers of the lake suggests that they are highly efficient, but unknown processes must operate to maintain the cell integrity.

Different types of adaptations to H_2_S have been defined among cyanobacteria based on the differential sensitivity of photosystems II and I to sulfide, and the capacity to carry out anoxygenic photosynthesis [76, 77]. However, the described processes can only function if a minimum intensity of light is available [78]. In dark and in anoxic conditions, cyanobacteria have the capacity to perform fermentative metabolism coupled with sulfur reduction [79]. Dark and anaerobic conditions have been shown to enhance the survival of *Oscillatoria terebreformis* compared with that under aerobic conditions [74].

The negative relationships between the abundance of the two taxa and NH_4_^+^ content probably originated from the significant increase in its concentration in the bottom waters. The observed values were among the highest recorded in aquatic ecosystems [80], and therefore, might be toxic or sub-toxic for both the taxa. Additionally, under high temperature and pH conditions observed in the lake, the ratio between toxic free ammonia (NH_3_) versus ammonium (NH_4_^+^) might increase, further affecting cell growth and viability [81].

Salinity has also been reported by the model as a significant explanatory variable with a potential negative effect on the abundance of *Picocystis* and *Arthrospira*. The regression coefficients were relatively high for *Picocystis*, suggesting the higher sensitivity of *Picocystis* to salinity variations in the water column. However, the field observations of different studies suggest that *P. salinarum* is more tolerant to high salinity values (10–300 psu) than *A. fusiformis* (20–70 psu) [53]. In a laboratory experiment, Kebede [82] studied the growth of *A. fusiformis* strains subjected to a wide range of salinity and different salt compositions. They found an inverse relationship between salinity and growth, although the growth rate was still positive at a salinity of 90 psu (Table S3).

The apparent high sensitivity of *P. salinarum* to salinity in Lake Dziani Dzaha might be attributed to the conjunction of several adverse conditions that probably did not co-exist in the other ecosystems, where this species has been observed, and that have not been experimentally tested simultaneously.

Considering the above results, we can hypothesize that the co-existence of these two taxa in the same environmental niche is based on different adaptive features they might have to cope with light-limitation and adverse environmental conditions. To the best of our knowledge, this is the first in situ example of the role that niche differentiation in the light spectrum can play to complete the “nutrient-load hypothesis” [68]. However, one can ask why the coexistence of these two taxa was not reported in other saline-alkaline lakes, with the exception of one crater lake investigated by Krienitz et al. [14]. These authors provided the most probable explanation for the few reports of *Picocystis* as a co-occurrent species of cyanobacteria, writing that “This tiny protist is difficult to recognize and distinguish in field samples and is probably often neglected or wrongly identified.” During this study, we had the opportunity to use flow cytometry, which is currently the most efficient tool to enumerate picophytoplanktonic cells [83] and we also benefited from *P. salinarum* isolates to calibrate FCM analyses.

Regarding the ecological consequences of this co-existence, the two species might sustain two different trophic networks owing to their different morphological traits. The small size of *P. salinarum* might support a microbial loop [84] in the surface layer of the lake. Not only bacteria but also protozoans that have been detected in the lake [59] might benefit from the presence of these small phytoplanktonic cells either as a source of organic matter for aerobic heterotrophic bacteria or as a prey for nanoflagellates or protozoa. In contrast, due to their large size *A. fusiformis* filaments cannot be ingested by protozoans. By sedimentation, these filamentous organisms might act as a biological CO_2_ pump [85] and as the main source of (i) organic matter in the detrital bacterial loop of the anoxic bottom layer of the lake and (ii) long-term carbon stock stored in the sediment of the lake.

The presence of intact *A. fusiformis* and *P. salinarum* cells in the deepest layers of the lake, although representing only a small fraction of the abundance observed in the upper layer, implies that a part of the population of these two phylogenetically and morphologically distinct taxa was able to persist in extremely adverse conditions (anoxia, high H_2_S and NH_4_^+^ concentration, and no light). It is probable that the physiological status of the upper- and lower-layer cells is different. In the future, the survival behavior and physiological status of cells from isolated strains of these two taxa under controlled conditions should be evaluated. Furthermore, the transcriptome and metabolome of the two taxa along the depth profile should also be analyzed in order to understand the changes in certain metabolic pathways as a response to such extreme environmental conditions.

In conclusion, due to its geographical isolation, low anthropogenic pressure, thalassohaline signature associated with extreme physico-chemical conditions with a thin euphotic layer, and absence of grazers, Lake Dziani Dzaha is an excellent model to study the composition and functioning of a “simple” microbial aquatic ecosystem. By analyzing the phytoplanktonic community of this lake through high-depth sequencing approach, we verified the hypothesis that this community exhibits a very low richness and diversity like in other isolated and extreme aquatic ecosystems. Two OTUs (*A. fusiformis* and *P. salinarum*) appeared as the sole true pelagic phytoplanktonic OTUs in this lake. Furthermore, they not only appeared to form the minimal community that can be encountered in a present or “primitive” aquatic ecosystem, but also co-existed as a stable co-dominant consortium*,* at least according to the data collected during our four campaigns. Our findings are different from what is proposed by the classical community ecology hypotheses, according to which, in Lake Dziani Dzaha, only one species should have dominated the ecosystem. The two phylogenetically distinct OTUs (Cyanobacteria and Chlorophyta) showed distinct functional traits (pigment composition and size) that can explain their co-existence in the euphotic layer. Because of adverse environmental conditions that prevail below the surface layer, both the taxa exhibited a significant decrease in their abundances. Their behavior in the aphotic and anoxic water layers should be explored, and it constitutes one of the interesting challenges for future studies.

## Electronic supplementary material

Table S1 Cyanobacterial OTU affiliation obtained by alignment of 16S rDNA sequences with NCBI BLAST tool, Genbank Databases, and sequences from strains isolated from Lake Dziani Dzaha [18]. (DOCX 16 kb)

Table S2 Plastid-related OTU affiliation obtained by alignment of 16S rDNA plastids sequences with NCBI BLAST tool, Genbank Databases, and plastids sequences from Phytoref [46]. (DOCX 17 kb)

Table S3 Review of *Arthrospira fusiformis* (Cyanobacteria) and *Picocystis salinarum* (Chlorophyta) characteristics. (DOCX 44.4 kb)

Fig. S1 Cyanobacterial OTUs affiliation using consensus maximum likelihood phylogenetic tree based on 16S rRNA gene sequences of representative Cyanobacteria strains isolated from Dziani Dzaha (Cellamare et al., 2018) and Genbank. Numbers above branches indicate bootstrap support (> 50%) from 1000 replicates. Bootstrap values are given in the following order: neighbor-joining/maximum likelihood/maximum parsimony (NL/ML/MP). The cyanobacterial OTUs from Dziani Dzaha are indicated in bold (OTUs 1, 10, 23, 60, 105 et 450). (DOCX 114 kb)

Fig. S2 Eukaryotic plastid-related OTUs affiliation using **c**onsensus Maximum Parsimony tree based on 16S rRNA gene sequences of photosynthetic-eukaryotes chloroplasts of representative sequences of Genbank and Phytoref [46]. Numbers above branches indicate bootstrap support (> 50%) from 1000 replicates. Bootstrap values are given in the following order: neighbor-joining/maximum likelihood/maximum parsimony (NL/ML/MP). OTUs from Lake Dziani Dzaha are indicated in bold (OTUs 2, 46, 115, 248 et 269). (DOCX 93 kb)

Fig. S3 Light microscope micrographs of **a** environmental sample from Lake Dziani Dzaha (DZ-15-11 campaign) with *Arthrospira fusiformis* straight filaments and unicellular cells of *Picocystis salinarum*, **b** culture of *Arthrospira fusiformis* strain (PMC 851.14), **c** culture of *Picocystis salinarum* strain (ALCP 144.1) © C. Duval, MNHN. Scale bar = 10 μm. (DOCX 2904 kb)

Fig. S4 Comparison of normalized sequences number with the cells abundance (cells mL^−1^) for the two dominant taxa: *Arthrospira fusiformis* and *Picocystis salinarum*. Data all the four campaigns (*n* = 28) have been plotted. (DOCX 20 kb)
