# Supplementary material for: Very Low Phytoplankton Diversity in a Tropical Saline-Alkaline Lake, with Co-dominance of Arthrospira fusiformis (Cyanobacteria) and Picocystis salinarum (Chlorophyta)
Source: Microb Ecol. 2019 Feb 7;78(3):603–17. doi: 10.1007/s00248-019-01332-8 (PMC6744573; doi:10.1007/s00248-019-01332-8)
Supplement: Supplementary file 1 — Cyanobacterial OTU affiliation obtained by alignment of 16S rDNA sequences with NCBI BLAST tool, Genbank Databases, and sequences from strains isolated from Lake Dziani Dzaha [18]. (DOCX 16 kb) [file 248_2019_1332_MOESM1_ESM.docx]

**Table S1** Cyanobacterial OTU affiliation obtained by alignment of 16S rDNA sequences with NCBI BLAST tool, Genbank Databases, and sequences from strains isolated from Lake Dziani Dzaha [18].

| **OTU**  **number** | **NCBI affiliation** | **Genbank affiliation** | **Identity (%)** | **PMC reference strains** | **Identity (%)** |
| --- | --- | --- | --- | --- | --- |
| **1** | *Arthrospira* | *Arthrospira platensis* | 99% | *Arthrospira fusiformis* | 99% |
| **10** | Unknown | *Leptolyngbya* | ≤ 95% | *Leptolyngbya* | ≤ 95% |
| **23** | *Lyngbya* | *Microcoleus / Geitlerinema* | 99% | *Sodalinema komarekii* | 99% |
| **60** | *Synechococcus* | *Synechococcus* | 99% | *Spirulina subsalsa* | ≤ 90% |
| **105** | *Xenococcus* | *Xenococcus* | 98% | *Spirulina subsalsa* | ≤ 92% |
| **450** | *filamentous cyanobacterium LLi71* | *filamentous cyanobacterium LLi71* | 99% | *Leptolyngbya* | ≤ 94% |
| **339** | *Oscillatoriales cyanobacterium* | *Leptolyngbya* | 97% | *Leptolyngbya* | ≤ 93% |
| **365** | *Oscillatoriales cyanobacterium* | *Leptolyngbya* | 97% | *Leptolyngbya* | ≤ 93% |
