# Supplementary material for: Very Low Phytoplankton Diversity in a Tropical Saline-Alkaline Lake, with Co-dominance of Arthrospira fusiformis (Cyanobacteria) and Picocystis salinarum (Chlorophyta)
Source: Microb Ecol. 2019 Feb 7;78(3):603–17. doi: 10.1007/s00248-019-01332-8 (PMC6744573; doi:10.1007/s00248-019-01332-8)
Supplement: Supplementary file 2 — Plastid-related OTU affiliation obtained by alignment of 16S rDNA plastids sequences with NCBI BLAST tool, Genbank Databases, and plastids sequences from Phytoref [46]. (DOCX 17 kb) [file 248_2019_1332_MOESM2_ESM.docx]

**Table S2** Plastid-related OTU affiliation obtained by alignment of 16S rDNA plastids sequences with NCBI BLAST tool, Genbank Databases, and plastids sequences from Phytoref [46].

| **OTU**  **number** | **NCBI affiliation** | **Genbank affiliation** | **Identity (%)** | **Phytoref affiliation** | **Identity (%)** |
| --- | --- | --- | --- | --- | --- |
| **2** | *Picocystis salinarum* | *Picocystis salinarum* | 99% | *Picocystis salinarum* | 99% |
| **115** | *Kryptoperidinium foliaceum* | *Nitzschia/Kryptoperidinum/Amphora* | 99% | *Cymbella* | 99% |
| **46** | Unknown | *Amphiprora/Planoglabratella* | 99% | *Cymbella/Cymbopleura* | 99% |
| **148** | Multi-affiliation | *Pinnularia/Planoglabratella* | 99% | *Cymbella fuegensis* | 99% |
| **248** | Unknown | *Eunotia/Haslea/Asterionellopsis* | 99% | *Cymbella/Haslea* | 99% |
| **269** | Unknown | *Planoglabratella/Navicula* | 98% | *Naviculacea* | 99% |
| **85** | *Gyrosigma fasciola* | *Gyrosigma fasciola* | 99% | *Gyrosigma fasciola* | 99% |
