# Supplementary material for: Very Low Phytoplankton Diversity in a Tropical Saline-Alkaline Lake, with Co-dominance of Arthrospira fusiformis (Cyanobacteria) and Picocystis salinarum (Chlorophyta)
Source: Microb Ecol. 2019 Feb 7;78(3):603–17. doi: 10.1007/s00248-019-01332-8 (PMC6744573; doi:10.1007/s00248-019-01332-8)
Supplement: Supplementary file 3 — Review of Arthrospira fusiformis (Cyanobacteria) and Picocystis salinarum (Chlorophyta) characteristics. (DOCX 44.4 kb) [file 248_2019_1332_MOESM3_ESM.docx]

**Table S3** Review of *Arthrospira* *fusiformis* (Cyanobacteria) and *Picocystis* *salinarum* (Chlorophyta) characteristics.

| **Descriptors** | ***Arthrospira* *fusiformis*** | ***Picocystis* *salinarum*** |
| --- | --- | --- |
| Phylum | Bacteria | Eukaryota |
| Synonymous | *Spirulina platensis, Arthrospira platensis* | - |
| Phylogeny | Cyanobacteria, Oscillatoriales, well supported cluster of *Arthrospira* [18] | Chlorophyta, Prasinophyceae, not well supported Clade VII [50]. |
| Habitats | Wide range distribution in freshwater to hypersaline habitats [86] | Widespread in saline to hypersaline ecosystems. Oxic and anoxic environments [86]. |
| Morphology | Planktonic, filamentous, solitary trichomes, lengthened (170-2,390 µm), cells are 9-12 μm wide, 3-7 µm length, with aerotopes regulating buoyancy, non heterocytous, generally coiled with coil diameter: 15-60 µm and distance between two coils: 0-80 µm but straight in Lake Dziani Dzaha, multiplication by fragmentation [18] | Planktonic, green, spherical or ovoid, about 1.5-2.0 µm in diameter, no flagella, bilobed chloroplast [18, 87] |
| Biovolume (µm^3^) | Cylindrical cell (this study): 235 | Spherical cell (this study): 10 |
| Surface/volume | Straight filament: 0.45 | Spherical cells: 2.2 |
| Sinking properties | Regulated by the numerous aerotopes and buoyancy, straight filament [18] | Low intrinsic sinking rate due to the small size [70] |
| Biomass | Highly productive primary producer. Max. 200-1430 mg L^-1^ dry mass | Highly productive primary producer. Max. 1.0-3.5 x 10^9^ cells L^-1^ [14] |
| Grazers | Lesser Flamingos, fish (*Alcolapia graham* Boulenger, rotifers (*Brachionus dimidiatus* Bryce), *B. plicatilis* Müller, *Hexarthra jenkinae* De Beauchamps), copepods (*Lovenula africana* Daday), insects (chironomids) [86]. | Brine shrimp: *Artemia monica* [73]. Crustacean: *Lovenula africana* Daday [88]. Rotifers: *Brachionus dimidiatus* Bryce and *B. plicatilis* Müller [14]. Amoebae. |
| Biomass crash causes | Cyanophages [89] | Unknown |
| Salinity tolerance | Natural conditions: 1-300 psu [90]  Laboratory conditions: 8.5 - 200 psu [91, 92] | Natural conditions: 10-300 psu. Able to outcompete *Arthrospira* in high salinity conditions [86].  Laboratory conditions: 0-260 psu [73] |
| Osmoprotectants | Accumulation of low MW carbohydrates: glucosyl-glycerol, trehalose, glucosylglycerate [93] | Cellular glycine betaine, dimethylsulfoniopropionate [73]. |
| Temperature tolerance | >35°C [94] | >30°C [95] |
| pH tolerance | >11 [96]  pH homeostasis dependent of sodium concentrations [97] | 4-12 [73] |
| Growth rate | Fast-growing : 0.8-1.5 d^-1^, at 35 °C, under saline conditions [98] | Fast-growing rate: 0.7-1 d^-1^ [14] |
| Pigments (specific wavelengths absorbance in nm) | Majors: Chlorophyll-a (431, 617, 666 nm), phycocyanin (550-650)  Minors: carotenoids β-carotene, zeaxanthin (this study). | Majors: Chlorophyll a (431, 617, 666 nm), Chlorophyll b (461, 599, 648 nm), β-carotene (454, 479 nm), diatoxanthin (451, 479 nm), monadoxanthin (448, 475 nm)  Minors: neoxanthin, violaxanthin, antheraxanthin, lutein, zeaxanthin, alloxanthin [73, 87, 99] |
| Adaptations to high turbidity | Buoyancy with aerotopes (gas vesicles), high cellular pigment concentrations | Positive growth at very low irradiance (e.g., 0.6 µmol photons m-^2^ s-^1^ |
| Adaptations to light | Adaptation to high light: self-shading, photoprotective mechanisms, accumulation of bicarbonate in the cytoplasm [93] | Adapted to low irradiance. Positive growth rate (0.2 d^-1^) at 0.6 µmol photons m^-2^ s^-1^ [73] |
| Proteins | 60-70 % per unit dry mass [100] |  |
| Lipids | Low content (depending of the culture conditions) | High content during nitrogen limitation [95] |
| Carbohydrates | 10 to 15 % of dry biomass, mainly as rhamnose and glycogen [93] |  |
| Genome size | 6 to 6.7 Mpb [93] | 25 Mbp estimated by flow cytometry [99] |
| N_2_-fixation potential | Non-nitrogen fixing species but genes responsible for heterocyst maturation (*patU, hetR, hetF*) and nitrogen fixation conserved in all *Arthrospira* genomes [93] | Non-nitrogen fixing species |

**References**

86. Krienitz L, Schagerl M (2016) Tiny and tough: microphytes of East African Soda Lakes

87. Lewini RA, Krienltz L, Oerickei RG, et al (2000) picoplanktonic green alga. 39:560–565

88. Vareschi E, Vareschi A (1984) The ecology of Lake Nakuru (Kenya). Oecologia 61:70–82. https://doi.org/10.1007/BF00379091

89. Peduzzi P, Gruber M, Gruber M, Schagerl M (2014) The virus’s tooth: cyanophages affect an African flamingo population in a bottom-up cascade. ISME J 8:1346–1351. https://doi.org/10.1038/ismej.2013.241

90. Dadheech N (2010) Desiccation tolerance in cyanobacteria. African J Microbiol Res 4:1584–1593

91. Arruda R, Brito A, da Silva R, Moraes I (2009) Fermentação De Spirulina Platensis Sob Condições Naturais De Temperatura E Insolação. Rev Saúde 3:16–19

92. Pelizer LH, Moraes IDO (2009) Development of solid state cultivation process for Spirulina platensis production. New Biotechnol. 25:S223–S224. https://doi.org/10.1016/j.nbt.2009.06.189

93. Furmaniak MA, Misztak AE, Franczuk MD, Wilmotte A, Waleron M, Waleron KF (2017) Edible cyanobacterial genus Arthrospira: actual state of the art in cultivation methods, genetics, and application in medicine. Front. Microbiol. 8:1–21. https://doi.org/10.3389/fmicb.2017.02541

94. Vonshak A, Tomaselli L (2000) Arthrospira (spirulina): systematics and ecophysioIogy. The ecology of cyanobacteria. Springer, Dordrecht, pp 505–522

95. Wang S, Lambert W, Giang S, Goericke R, Palenik B (2014) Microalgal assemblages in a poikilohaline pond. J. Phycol. 50:303–309. https://doi.org/10.1111/jpy.12158

96. Belkin S, Boussiba S (1991) Resistance of Spirulina platensis to ammonia at high pH values. Plant Cell Physiol 32:953–958. https://doi.org/10.1093/oxfordjournals.pcp.a078182

97. Schlesinger WH, Raikes JA, Hartley AE, Cross AF (1996) On the spatial pattern of soil nutrients in desert ecosystems. Ecology 77:364–374. https://doi.org/10.2307/2265615

98. Vonshak A (1997) Spirulina platensis (Arthrospira): physiology, cell-biology, and biotechnology. Taylor & Francis, London

99. Lopes Dos Santos A, Pollina T, Gourvil P et al (2017) Chloropicophyceae, a new class of picophytoplanktonic prasinophytes. Sci. Rep. 7:1–20. https://doi.org/10.1038/s41598-017-12412-5

100. Belay A (2002) The potential application of Spirulina (Arthrospira) as a nutritional and therapeutic supplement in health management
