# Supplementary material for: Very Low Phytoplankton Diversity in a Tropical Saline-Alkaline Lake, with Co-dominance of Arthrospira fusiformis (Cyanobacteria) and Picocystis salinarum (Chlorophyta)
Source: Microb Ecol. 2019 Feb 7;78(3):603–17. doi: 10.1007/s00248-019-01332-8 (PMC6744573; doi:10.1007/s00248-019-01332-8)
Supplement: Supplementary file 4 — Cyanobacterial OTUs affiliation using consensus maximum likelihood phylogenetic tree based on 16S rRNA gene sequences of representative Cyanobacteria strains isolated from Dziani Dzaha (Cellamare et al., 2018) and Genbank. Numbers above branches indicate bootstrap support (> 50%) from 1000 replicates. Bootstrap values are given in the following order: neighbor-joining/maximum likelihood/maximum parsimony (NL/ML/MP). The cyanobacterial OTUs from Dziani Dzaha are indicated in bold (OTUs 1, 10, 23, 60, 105 et 450). (DOCX 114 kb) [file 248_2019_1332_MOESM4_ESM.docx]

**Fig. S1**

0.050

***Sodalinema komarekii***

**OTU 23**

*Sodalinema komarekii* (PMC 865.14)

*Oscillatoria* sp. SAG 76.79 (NCBI nr) (KM019969.1)

*Geitlerinema* sp. CCY0102 (NCBI nr) (GQ402015.1)

*Geitlerinema* sp. BBD (78064498 DQ151461.1)

*Arthrospira platensis* (NIES39 88792645 DQ393279.1) (PCC 7345 672239122 NR 125711.1)

***Arthrospira fusiformis***

**OTU 1**

*Arthrospira fusiformis* (PMC 738.11)

*Arthrospira maxima* str. Lefevre (1963/M1321 226525447 FJ798612.1) (KCTC AG30054 DQ393281.1)

*Arthrospira fusiformis* (AB2002/01 AY575923.1) (AB2002/02 AY575924.1)

*Arthrospira indica* (PD2002/ana AY575932.1) (PD1998/pus AY575930.1)

*Desertifilum tharense* PD2001/TDC7 (224995585 FJ158995.1) (FJ158997.1)

*Desertifilum fontinale* KR2012/2 (Dadheech) (689455817 KJ028038.1)

*Desertifilum diaznensis* (PMC 872.14)

***Synechococcus* sp.**

**OTU 60**

*Synechococcus* sp. PCC 8806 (NCBI nr) (AF448077.1)

*Spirulina subsalsa* (PMC 857.14)

*Spirulina subsalsa* AB2002/06 (50428832 AY575934.1) (AY575935.1) (498424979 HF678502.1)

*Xenococcus* sp. RCC2703 (NCBI) (KT861357.1) (KT861356.1)

***Xenococcus* sp.**

**OTU 105**

*Xenococcus* sp. CR L15 (NCBI nr) (EF545631.1) (EF545606.1) (EF545618.1)

*Xenococcus* sp. PFBA1 (NCBI) (KF650431.1)

*Leptolyngbya tenuis* PMC304.07 (306031907 GQ859652.1)

*Leptolyngbya boryana* PCC 6306 (148529697 EF429290.1) (84453006 AB245143.1)

*Sodalyngbya stromatolii* (PMC 867.14)

*Oscillatoria acuminata* PCC 6304 (664803865 KM019978.1)

*Oscillatoriales cyanobacterium* UVFP2 (NCBI nr) (AJ630648.1)

*Leptolyngbya* sp. LEGE 07089 (NCBI nr) (HM217063.1)

**OTU 339**

**OTU 365**

***Leptolyngbya* sp. *3 & 4***

*Leptolyngbya augustissima* SABC030403 (named as *Phormidium angustissimum*) (NCBI) (KX818203.1)

***Leptolyngbya* sp. *2***

**OTU 450**

Filamentous cyanobacterium LLi71 (NCBI nr) (DQ786167.1)

***Leptolyngbya* sp. *1***

**OTU 10**

*Haloleptolyngbya alcalis* (PMC 892.15)

*Haloleptolyngbya alcalis* KR2005/106 (Dadheech) (383234783 JN712770.1)

*Leptolyngbya cf. halophila* LEGE 06152 (318101613 HQ832915.1)

*Leptolyngbya* sp.(PMC 895.15)

*Leptolyngbya* sp. 0BB19S12 (82697091 AJ639895.1)

*Leptolyngbya foveolarum* PMC302.07 (306031908 GQ859653.1)

*Rhodopseudomonas palustris* strain ATCC 17001 (NCBI outgroup) (NR 115484.1)

100/100/100

100/99/100

100/99/99

92/94/96

100/100/100

85/80/75

99/96/99

-/51/60

97/98/99

-/53/60

100/98/99

95/89/87

100/98/98

97/91/90

51/62/78

99/92/98

64/51/67

99/82/100

95/87/95

90/88/93

96/88/90

88/62/-
