# Supplementary figures and images for: Very Low Phytoplankton Diversity in a Tropical Saline-Alkaline Lake, with Co-dominance of Arthrospira fusiformis (Cyanobacteria) and Picocystis salinarum (Chlorophyta)
Source: Microb Ecol. 2019 Feb 7;78(3):603–17. doi: 10.1007/s00248-019-01332-8 (PMC6744573; doi:10.1007/s00248-019-01332-8)

**Fig. S3**


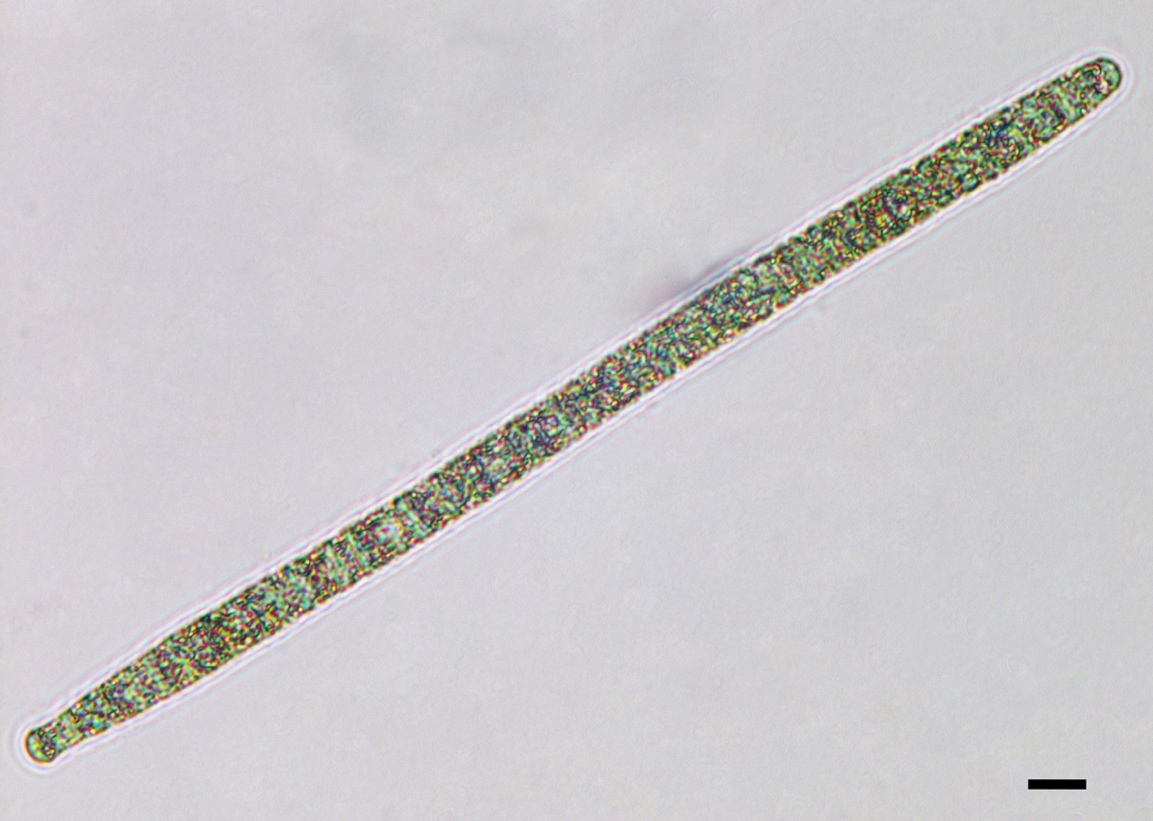

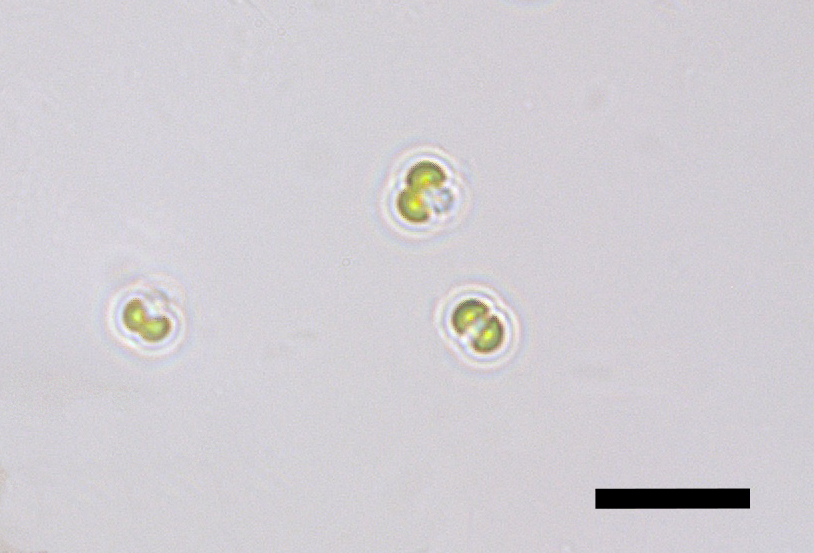

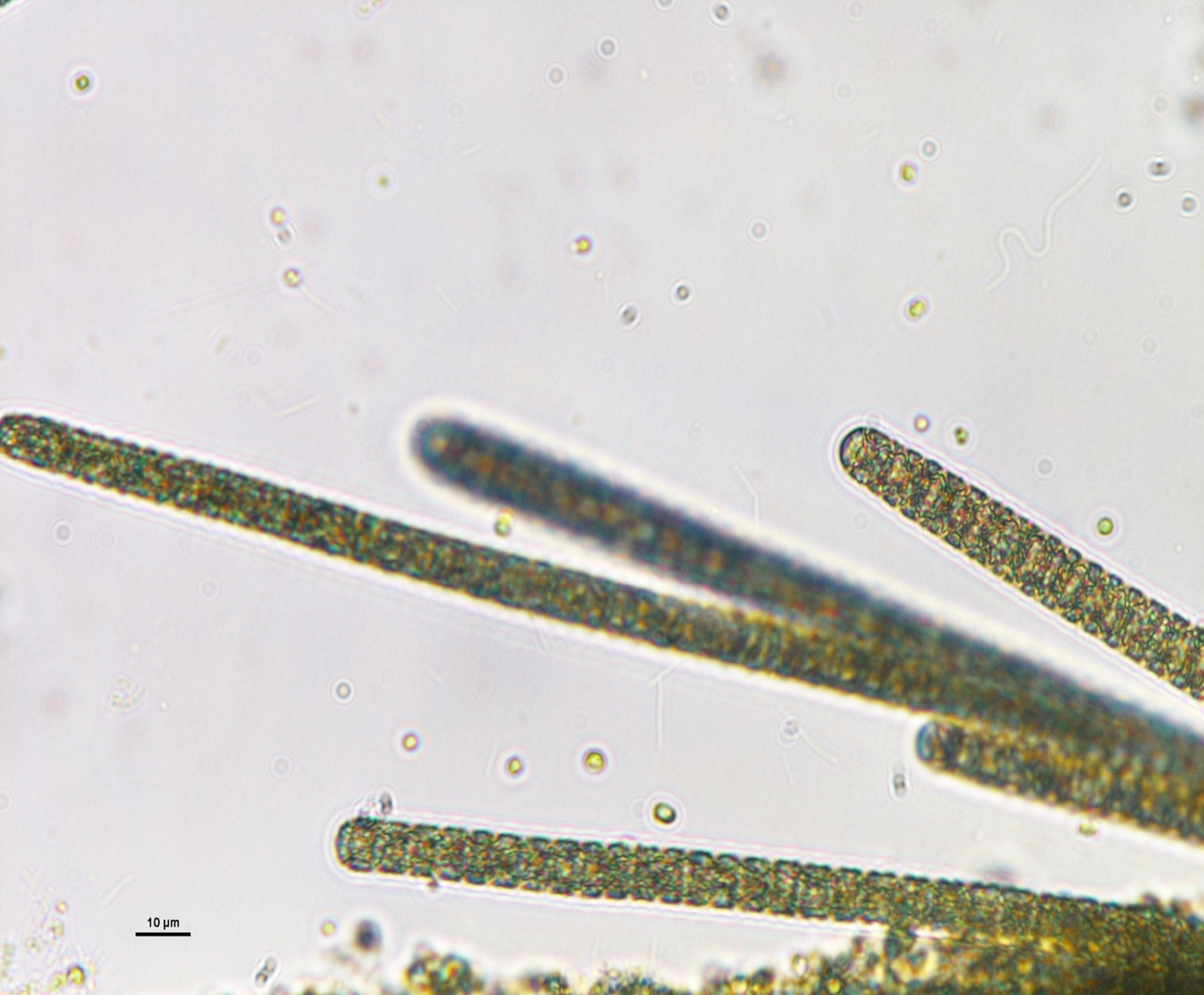


A

B

C

Supplement: Supplementary file 6 — Light microscope micrographs of a environmental sample from Lake Dziani Dzaha (DZ-15-11 campaign) with Arthrospira fusiformis straight filaments and unicellular cells of Picocystis salinarum, b culture of Arthrospira fusiformis strain (PMC 851.14), c culture of Picocystis salinarum strain (ALCP 144.1) © C. Duval, MNHN. Scale bar = 10 μm. (DOCX 2904 kb) [file 248_2019_1332_MOESM6_ESM.docx]
